# CD4+ and Perivascular Foxp3+ T Cells in Glioma Correlate with Angiogenesis and Tumor Progression

**DOI:** 10.3389/fimmu.2017.01451

**Published:** 2017-11-07

**Authors:** Luyan Mu, Changlin Yang, Qiang Gao, Yu Long, Haitao Ge, Gabriel DeLeon, Linchun Jin, Yifan (Emily) Chang, Elias J. Sayour, Jingjing Ji, Jie Jiang, Paul S. Kubilis, Jiping Qi, Yunhe Gu, Jiabin Wang, Yuwen Song, Duane A. Mitchell, Zhiguo Lin, Jianping Huang

**Affiliations:** ^1^Department of Neurosurgery, The Fourth Affiliated Hospital, Harbin Medical University, Harbin, China; ^2^The Fourth Section of Department of Neurosurgery, The First Affiliated Hospital, Harbin Medical University, Harbin, China; ^3^Lillian S. Wells Department of Neurosurgery, University of Florida, Gainesville, FL, United States; ^4^Department of Geriatrics, The Second Affiliated Hospital of Harbin Medical University, Harbin, China; ^5^Department of Pathology, The Second Affiliated Hospital of Harbin Medical University, Harbin, China; ^6^Department of Pathology, The First Affiliated Hospital, Harbin Medical University, Harbin, China

**Keywords:** gliomas, angiogenesis, tumor-infiltrating lymphocytes, progression, recurrence

## Abstract

**Background:**

Angiogenesis and immune cell infiltration are key features of gliomas and their manipulation of the microenvironment, but their prognostic significance remains indeterminate. We evaluate the interconnection between tumor-infiltrating lymphocyte (TIL) and tumor blood-vasculatures in the context of glioma progression.

**Methods:**

Paired tumor tissues of 44 patients from three tumor-recurrent groups: diffuse astrocytomas (DA) recurred as DA, DA recurred as glioblastomas (GBM), and GBM recurred as GBM were evaluated by genetic analysis, immunohistochemistry for tumor blood vessel density, TIL subsets, and clinical outcomes. These cells were geographically divided into perivascular and intratumoral TILs. Associations were examined between these TILs, CD34+ tumor blood vessels, and clinical outcomes. To determine key changes in TIL subsets, microarray data of 15-paired tumors from patients who failed antiangiogenic therapy- bevacizumab, and 16-paired tumors from chemo-naïve recurrent GBM were also evaluated and compared.

**Results:**

Upon recurrence in primary gliomas, similar kinetic changes were found between tumor blood vessels and each TIL subset in all groups, but only CD4+ including Foxp3+ TILs, positively correlated with the density of tumor blood vessels. CD4 was the predominant T cell population based on the expression of gene-transcripts in primary GBMs, and increased activated CD4+ T cells were revealed in Bevacizumab-resistant recurrent tumors (not in chemo-naïve recurrent tumors). Among these TILs, 2/3 of them were found in the perivascular niche; Foxp3+ T cells in these niches not only correlated with the tumor vessels but were also an independent predictor of shortened recurrence-free survival (RFS) (HR = 4.199, 95% CI 1.522–11.584, *p* = 0.006).

**Conclusion:**

The minimal intratumoral T cell infiltration and low detection of CD8 transcripts expression in primary GBMs can potentially limit antitumor response. CD4+ and perivascular Foxp3+ TILs associate with tumor angiogenesis and tumor progression in glioma patients. Our results suggest that combining antiangiogenic agents with immunotherapeutic approaches may help improve the antitumor efficacy for patients with malignant gliomas.

## Introduction

Tumors recur in the resection margin in nearly 90% of CNS malignancies after primary surgery and adjuvant therapies (1, 2). Understanding which significant alterations occur in the tumor immune microenvironment during progression is critical for designing effective immunotherapeutic strategies. Tumor angiogenesis and immune cell infiltrations are crucial tumor-driven processes in tumors (3, 4); however, mechanistic insights regarding their interplay, as well as their prognostic significance in glioma recurrence remain indeterminate.

Astrocytic gliomas arise from astrocytes and are the most common type of glioma, representing 64% of human CNS malignant tumors (5). Nearly all the patients with primary gliomas will face tumor recurrence thus necessitating a better understanding of glioma progression. Recently, the angiogenesis inhibitor Bevacizumab has been shown to prolong recurrence-free survival (RFS), although no increase in overall survival (OS) was seen, in patients with newly diagnosed or recurrent GBM (6–8). This drug inhibits the formation of tumor blood vessels induced by vascular endothelial growth factor A (VEGF-A); increases T cell infiltrations (9, 10); reverses expression of inhibitory molecules associated with T cell exhaustion (11); and may directly mediate antitumor effect (12). These results imply that angiogenesis plays an important role in GBM progression, but the failure of these agents to improve patients’ OS also suggest that negative feedback pathways (such as hypoxia) may be spontaneously activated resulting in increased tumor cell invasion (13, 14).

Tumors can orchestrate complex biological networks via angiogenesis and recruitment of regulatory immune cell subsets. The VEGF/receptor axis has been shown to have strong immune regulatory properties (3, 9–11). Under hypoxia condition, tumor-infiltrating lymphocytes (TILs) were found to express VEGF-A (15). Evidence indicates that the tumor blood vessel/tumor endothelium can be a substantial barrier for TIL to extravasate into the intratumoral space stymieing their ability to mount a strong antitumor response (16). Distinctly separating TILs into perivascular and intratumoral populations will provide better insight for us to determine the interaction between tumor angiogenesis and T cell infiltration.

Although several studies have previously evaluated lymphocyte infiltrations in glioma patients (17–22), those studies have focused on non-paired patient samples, which could potentially introduce significant variations. Since gliomas are heterogeneous, district genetic or phenotypic characteristics have been observed in cells from the same tumor (23, 24), which may be conserved in recurrence. Distinctions between primary and recurrent tumors in the same patient may enable a better understanding of transition/transformation during tumor progression. In this study, we studied 44 paired tumor samples (before and after of progression) in three categories of recurrence from astrocytic glioma patients and probed the interconnection between differential geographic T cell subsets and tumor blood vessels. Additionally, we also analyzed 15-paired GBMs samples from patients who had failed bevacizumab therapy and determined distinct TIL changes within these tumors. Our data suggest that CD4+ and perivascular Foxp3+ TILs impact angiogenesis and tumor recurrence in patients with gliomas.

## Materials and Methods

### Patient Population

This study included 44 patients who underwent surgery for primary and recurrent glioma by MRI imaging at the same hospital from 2005 to 2014 (Table S2 in Supplementary Material). The average period between diagnosis and surgeries was 5.4 days (range 2–10 days). The inclusion criteria included the following: greater than 18 years of age at the time of primary diagnosis, a histopathological diagnosis of diffuse astrocytomas (DA) or GBM, and the availability of formalin-fixed, paraffin-embedded (FFPE) tumor tissue blocks. The patients were separated into three groups: (1) the DA–DA (DA, diffuse astrocytoma) group (DAs that recur as DAs, 15 pairs); (2) the GBM–GBM group (GBMs that recur as GBMs, 15 pairs); and (3) the DA–GBM group (DAs that recur as GBMs, 14 pairs). None of the 44 patients received treatments before their primary surgery. Subsequently, patients received standard radiotherapy (60 Gy), while some received concomitant–adjuvant chemotherapy (temozolomide/other). The recurrence-free survival (RFS) was defined as the time from the date of the primary surgery to the first MRI-confirmed tumor recurrence. All recurrent patients underwent recurrent surgery. OS was defined as the interval between primary surgery and death or last follow-up. Survival after recurrence (SR) was defined from the recurrent surgery to the date of death or the last follow-up. The mean follow-up period was 1,355.1 days (range 289–3,520 days), during which 36 patients died, and 8 patients remained alive. No patients were lost to follow-up. Tumor size was calculated based on enhanced MR or CT images (on the layer with the maximum amount of tumor) as follows: long diameter (centimeter) × wide diameter (centimeter) × thickness (centimeter) × 0.5 (25). All specimens were selected after histopathological review to ensure that we would have sufficient tissue for analysis. Treatment information was obtained from the time of primary surgery, including any adjuvant treatment. The latest updated OS information was obtained on February 13, 2016. For patients who were still alive, the date of the last follow-up was used in the data analysis. All subjects gave written informed consent in accordance with the Declaration of Helsinki and research protocols were reviewed and approved by the Committee on Human Research at the Harbin Medical University, China.

### Microarray Analysis of Gene Expression

To generate high-quality RNA from paired FFPE samples, a Sensation Plus™ FFPE Amplification and WT Labeling Kit was used to extract and amplify total RNA that was derived from whole tumor FFPE samples. The total RNA obtained from each sample was quantified using a NanoDrop ND-1000, and we selected three of the highest-quality paired samples from each group. The samples were used for labeling and submitted for hybridization array scanning using an Affymetrix Gene Chip^®^ Human Transcriptome Array 2.0. The microarray was processed, and the data were analyzed by Beijing Biolancet Technology, Co., Ltd. Nine pairs of samples were processed by Affymetrix HTA2.0 kit. After sequencing and QC, raw microarray data (CEL) were analyzed by Affymetrix Expression Console 1.4 software. HTA-2_0 library and annotation files were downloaded from Expression Console’s build-in database. Raw data were further normalized by the RMA-algorithm (Table S3 in Supplementary Material). Differentially expressed genes between samples were identified using fold-change filtering to identify distinct gene expression profiles between samples. For gene enrichment analysis, paired recurrent and primary gliomas samples’ microarray data were normalized by the log2 algorithm of genes mean value. Then, Gene Set Enrichment Analysis (GSEA) (26, 27) was applied to analyze the normalized data by groups. Enrichment Score, Normalized Enrichment Score, False Discovery Rate, and Nominal *p*-Value were reported by GSEA. Interpreting GSEA Results of the above four key statistics can be found in http://software.broadinstitute.org/gsea/doc/GSEAUserGuideFrame.html.

### Histological Analysis, Immunohistochemistry, and Immunofluorescence

A systematic neuro-pathological review was performed based on the 2007 World Health Organization classification guidelines for CNS tumors (28). Paired samples of slides were reviewed to determine their histological classifications. Immunohistochemical staining was performed for CD3 (clone LN10, dilution 1:200, Quanhui, China), CD4 (clone UMAB64, dilution 1:50; ZSGB-BIO, China), CD8 (clone SP16, dilution 1:100; ZSGB-BIO, China), Foxp3 (clone mAbcam 450, dilution 1:50; Abcam), and CD34 (clone EP88, dilution 1:150; ZSGB-BIO, China). The secondary antibodies came as an immunohistochemical kit (KIT-5930, Maxim, China) and were incubated for 40 min at room temperature. Each staining batch included a negative control that was processed without the primary antibody, a biological negative control that consisted of normal brain tissues (the donor had died of myocardial infarction), and a positive control (tonsil tissues).

The IHC analyses were performed using a quantitative approach under a light microscope (Leica SP2, Leica Optical Co. Ltd., Germany). Subpopulations of TILs expressing CD3, CD4, Foxp3, or CD8 were divided into intratumoral fractions, which contained no vessels in each high powered field (HPF) (40× objective and 10× eyepiece), and perivascular fraction, which contained more than one vessel in each HPF (29, 30). The mean counts of CD34+ vascular circles were obtained from 10 consecutive HPF by three pathologists blinded to outcome data. Similarly, for TIL frequencies, the results were determined from a mean of 10 consecutive HPFs of typical tumor regions. The average scores counted by three experienced pathologists blinded to the clinical background were recorded as the final result. When there was a large difference in scores between observers, the score was re-evaluated to reach an agreement. Results from CD3+, CD4+, FoxP3+, CD8+ cell, and CD34+ vascular circle counts in each patient were used in the statistical analysis.

The following antibodies were used for two-color immunofluorescence: rabbit anti-CD34 and mouse anti-CD3 monoclonal antibodies (as described above). The primary antibodies were incubated overnight at 4°C. The sections were then incubated with goat anti-mouse fluorescein-isothiocyanate-conjugated secondary antibodies and goat anti-rabbit rhodamine-conjugated secondary antibodies for 2 h at room temperature. The nuclei were stained with DAPI. Finally, the sections were visualized using a confocal microscope (as described above).

### Statistical Analysis

The raw data were tabulated using Microsoft Excel (Microsoft Corp.) and analyzed using nonparametric tests and matched-paired-tests (Wilcoxon tests or Mann–Whitney *U* tests). Chi-square tests were used to identify differences in chemotherapy and sex. Spearman’s rank order correlation analyses were performed to detect significant associations with positive marker expression. A Kaplan–Meier survival analysis was used to evaluate differences in RFS, SR, and OS. To adjust for potential confounders, Cox proportional hazards models were used to evaluate hazard ratios (HRs) for recurrence or death according to the number of identified TIL subpopulations and clinical features. All statistical analyses were performed using SPSS 13.0 (SPSS Inc. Chicago, IL, USA) and GraphPad Prism 6.0 (GraphPad Software Inc., La Jolla, CA, USA). All tests used to determine the level of significance were two-sided. A (two-tailed) *p*-value threshold of 0.05 was considered to indicate statistical significance (*p < 0.05, **p < 0.01, ***p < 0.001).

## Results

### Similar Kinetic Changes between Tumor-Associated Blood Vessels and TIL Subsets upon Recurrence

**Figure 1:**
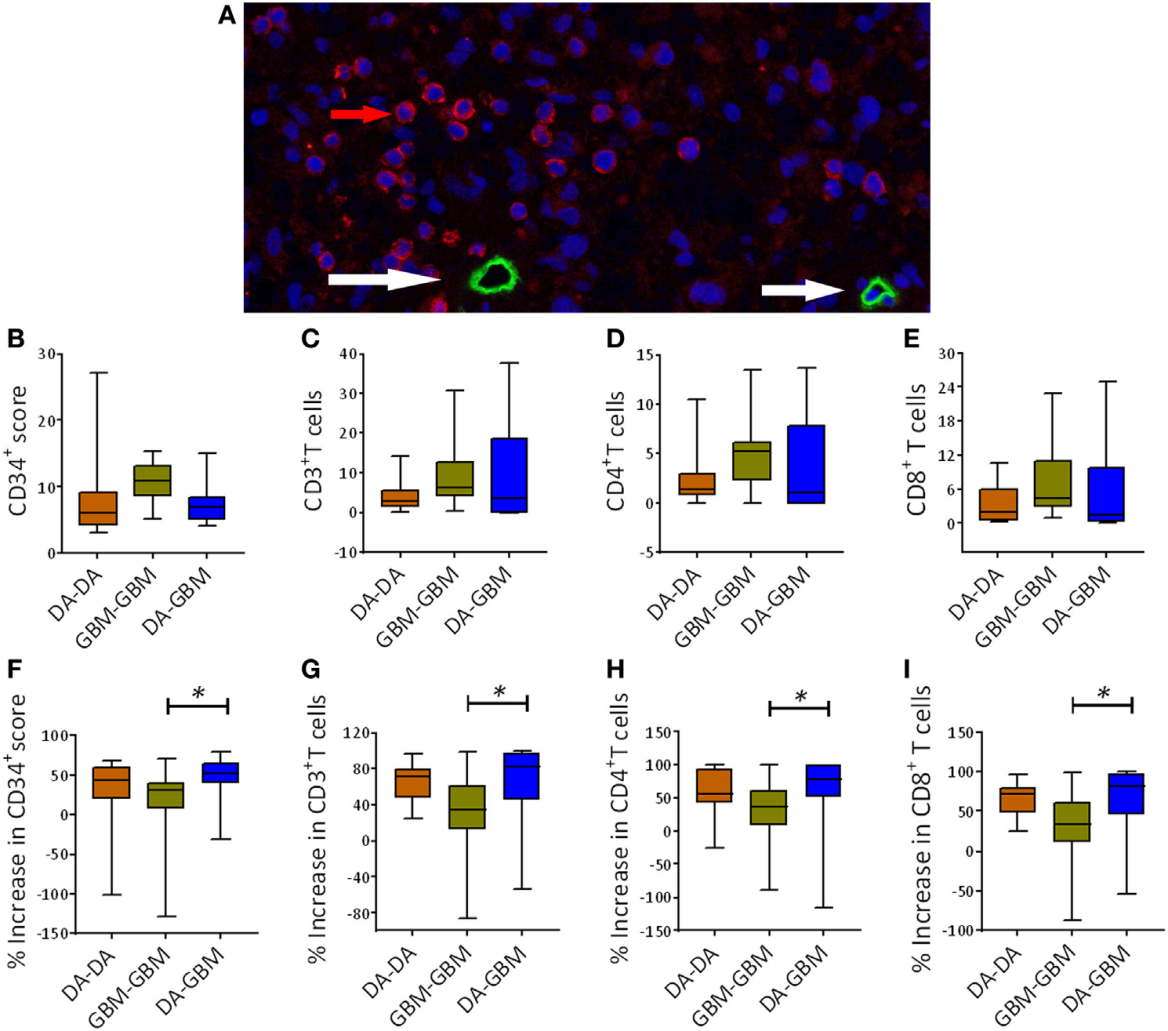
A similar kinetic movement of tumor angiogenesis and T cell infiltrations upon tumor recurrence [e.g., diffuse astrocytomas (DA)–DA, 15 pairs, DA–GBM, 15 pairs and GBM-GBM, 14 pairs]. **(A)** A representative fluorescent immunohistochemistry staining of tumor blood vessels measured by CD34 staining (green, red arrow) and tumor-infiltrating CD3+ T cells (red, white arrow) in a surgical resected primary GBM sample. **(B–E)** The baseline counts of CD34+ circles, CD3+, CD4+, and CD8+ cells in tumors. Counts were carried out for average numbers [10 consecutive high powered fields (HPFs)] of these marker expressing cells in primary tumors among three groups. **(F)** Enhancement of CD34+ circles after the recurrence. A calculation was carried out by comparing the increase in CD34+ circles between recurrent and primary tumors. **(G–I)** A similar measurement was also carried out for the average numbers of infiltrating CD3+, CD4+, and CD8+ T cells in these patients’ tumors, respectively. The significance between two groups was measured using Mann–Whitney *U* test, *p < 0.05.

To learn more about overall genetic changes before and after recurrence across the three recurrent groups (DA–DA, DA–GBM, and GBM-GBM), three pairs of FFPE glioma samples from each group were analyzed by Microarray. A distinct pattern of gene expression was observed before and after each recurrence (Figure S1A in Supplementary Material). Among these altered genes upon recurrence, VEGF-A was found increased by 10.2-fold after tumor recurrence in the DA–GBM recurrent group (Table S1 in Supplementary Material). Distinct clinical outcomes were also revealed in the three groups; the longest RFS was the DA to GBM recurrence (Table S2 and Figure S1B in Supplementary Material). Based on these results, blood vessels were measured using a vascular endothelial cell marker, CD34. Tumor-infiltrating T cells, such as CD3+, CD4+, and CD8+ T cells were determined by immunohistochemistry (Figure 1A; Figure S2A–C in Supplementary Material). Compared to other two groups, the primary tumors in the GBM-GBM group showed relatively higher on the average density of CD34+ circles and T cells, while the DA–GBM group displayed a wider-range of T cell infiltrations (Figures 1B–E). Similar kinetics of CD34+ circles and infiltrating T cells were observed when these parameters were compared before and after tumor recurrence/progression; compared to recurrent GBM, significantly increased CD34+ circles and T cell subsets were found in secondary GBM (Figures 1F–I).

### CD4+ T Cells Are Associated with Tumor Blood Vessels

**Figure 2:**
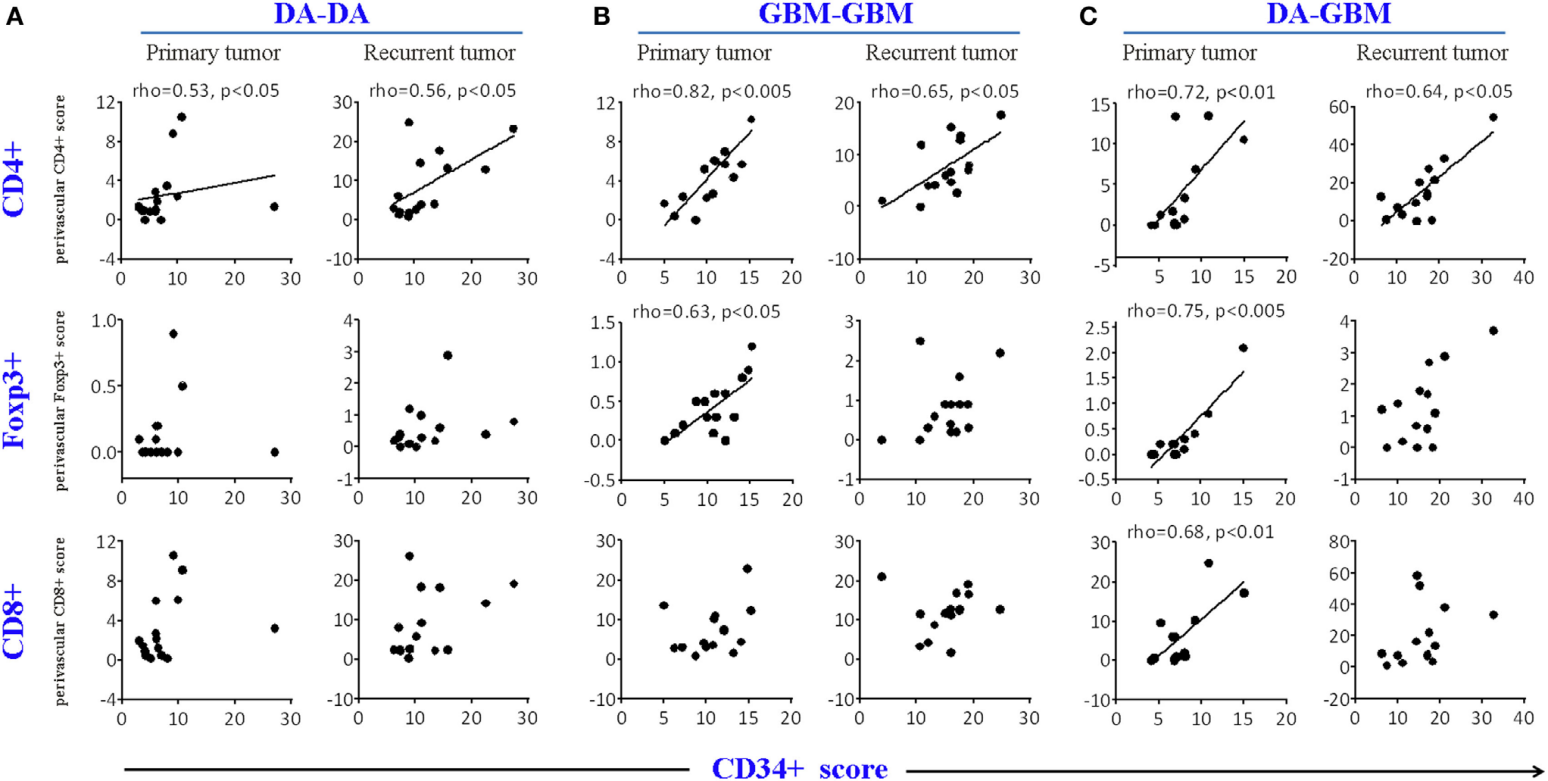
A correlation was found between the density of CD34 circles and CD4+, but not for CD8+ T cells, in primary and recurrent tumors. The correlation CD4+ or CD8+ T cell counts with CD34 circles [average cell or vascular circle counts were obtained from 10 consecutive high powered fields (HPFs)] in primary or recurrent tumors in diffuse astrocytomas (DA)–DA group **(A)**, GBM–GBM group **(B)**, and DA–GBM group **(C)**. Spearman’s rank order correlation analyses were performed and graphs with a correlation (Spearman’s rho ≥ 0.5, *p* < 0.05) are indicated.

To test which infiltrating T cell subset associated most closely with tumor angiogenesis, we next did a Spearman’s rank correlation analysis between the density of tumor vessels and T cell infiltrating subsets. CD4+ T cells correlated strongly with CD34+ circles in both primary and recurrent tumors across all three groups; notwithstanding primary tumors of DA–GBM, there was no association with CD8+ T cells (Figures 2A–C).

### CD4 Transcript Expression Is Abundant in Primary GBMs, and Activated CD4+ T Cells Are Enriched in Bevacizumab Resistant Tumors

**Figure 3:**
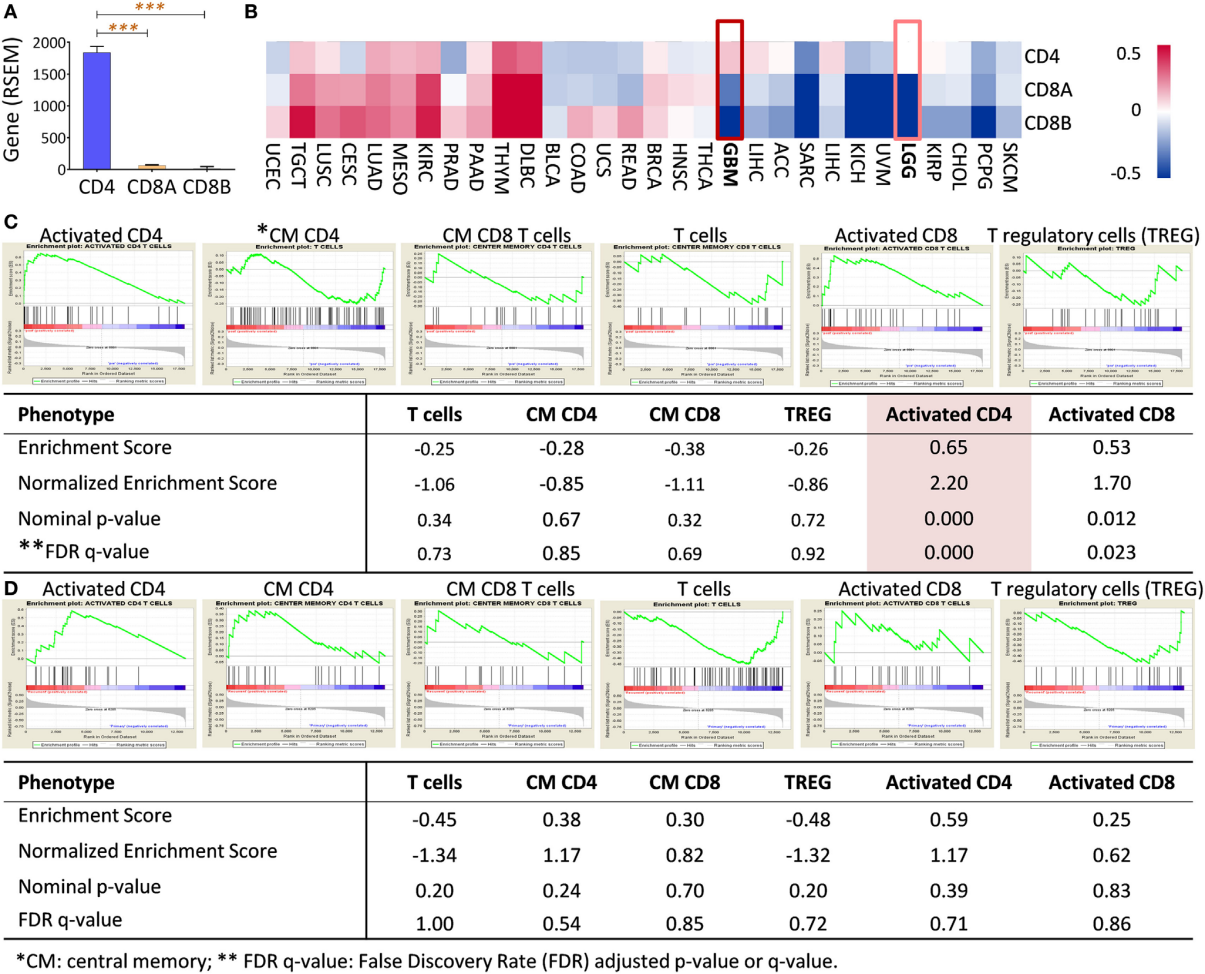
CD4 full-length transcript expression is abundant in primary GBM and enriched in bevacizumab-resistant tumors. **(A)** CD4 was a predominant gene transcript product (measured by RNA-seq by Expectation-Maximization, RSEM) of T cells in primary GBM compared with transcripts of CD8 alpha (CD8A) and beta (CD8B). **(B)** Gene profile of CD4, CD8A, and CD8B in other tumors, including GBM. RNAseq data of TCGA pan-cancer gene expression were used, and CD4, CD8A, and CD8B gene expressions from primary tumors were clustered as Heatmap using Subio Platform after logarithmic transformation and normalization. **(C)** Activated CD4+ T cells (highlighted) were significantly enriched in recurrent tumors after Bevacizumab treatment had failed in GBM patients. Paired surgical tumor samples before and after Bevacizumab treatment derived from 15 patients were analyzed by microarray (32). **(D)** No CD4 enrichment was in paired GBMs without any treatment. Microarray data from 16 pairs of tumor samples from recurrent GBM without any therapy were analyzed (33). The raw data were enriched between baseline and recurrent tumors using Gene Set Enrichment Analysis (GSEA) software (Broad Institute). The classification of the cell populations was based on the T cell-related metagenes (26).

We found that CD4 transcript was predominantly expressed in primary GBM (>29-fold higher than CD8 alpha and beta chains), based on the RNA-seq analysis solely culled from TCGA database (TCGA Research Network: http://cancergenome.nih.gov/, Figure 3A). No similar results were observed for some few other cancer types, such as kidney renal clear cell, testicular germ cell tumor using TCGA Pan-Cancer datasets (30 different cancer types) (Figure 3B). To confirm our observation that infiltrating CD4+ T cells may be associated with tumor angiogenesis, microarray data of paired bevacizumab resistant tumors from previously published reports were analyzed and paired chemo-naïve tumors were used as a comparison control (31, 32). Only signals of activated CD4+ T cells, not other cell populations, were significantly increased in tumors after a recurrence of bevacizumab-treated patients (Figure 3C); no such trend found in the chemo-naïve recurrent patient tumors (Figure 3D).

### Increasing Levels of Perivascular TILs after Glioma Recurrence

**Figure 4:**
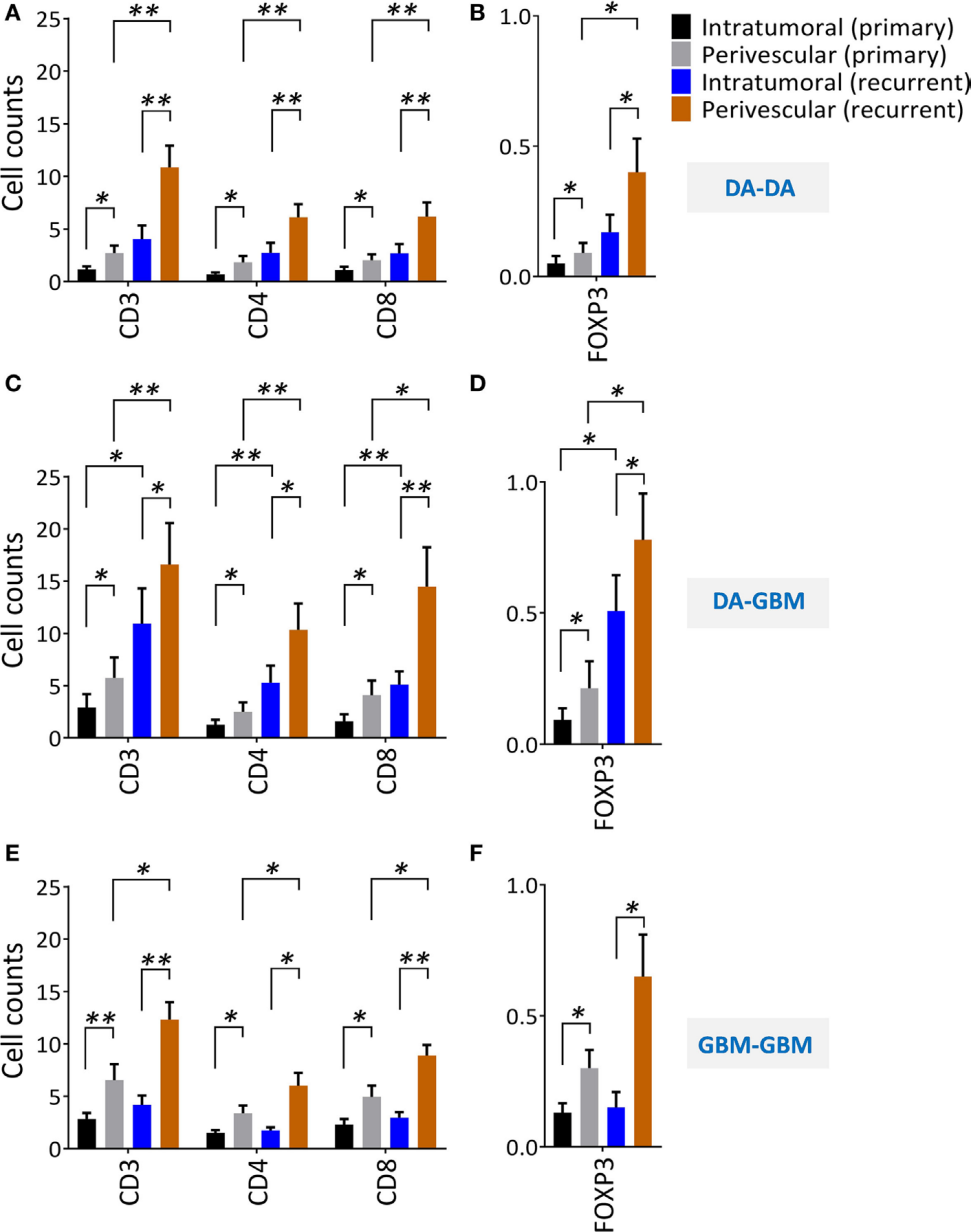
A significant increase of perivascular tumor-infiltrating lymphocytes after tumor recurrence. Perivascular and intratumoral infiltrating CD3+, CD4+, CD8+, and Foxp3+ T cells before and after recurrence were evaluated from randomly selected consecutive 10 high powered fields (HPFs). **(A,B)** diffuse astrocytomas (DA)–DA, **(C,D)** DA–GBM, and **(E,F)** GBM–GBM. Same scales are plotted for all the recurrent groups. The significances were determined respectively using paired *t*-test.

T cell tumor trafficking and tumor angiogenesis have been shown to be linked biological phenomena (3). We found both factors had an upwards trend upon recurrence; we next separated tumor-infiltrating T cells into perivascular and intratumoral CD3+, CD4+, CD8+, and Foxp3+ T cell subsets (Figure S2A–C in Supplementary Material). We demonstrated that there were more perivascular than intratumoral TILs in primary and recurrent tumors. In primary tumors, only one-third of the TILs were located in the intratumoral space, and two-thirds of the TILs were in the perivascular niche; these findings reproduce previously published reports (30). When comparing the cell numbers before and after recurrence, the perivascular T cells were enriched after tumor progression across all three groups; the intratumoral T cells, however, were only increased in recurrent tumors of the DA–GBM group (Figures 4A–F).

### Perivascular Foxp3+ Tumor-Infiltrating T Cells Associate with Angiogenesis and Is an Independent Factor That Predicts Glioma Progression/Recurrence

**Figure 5:**
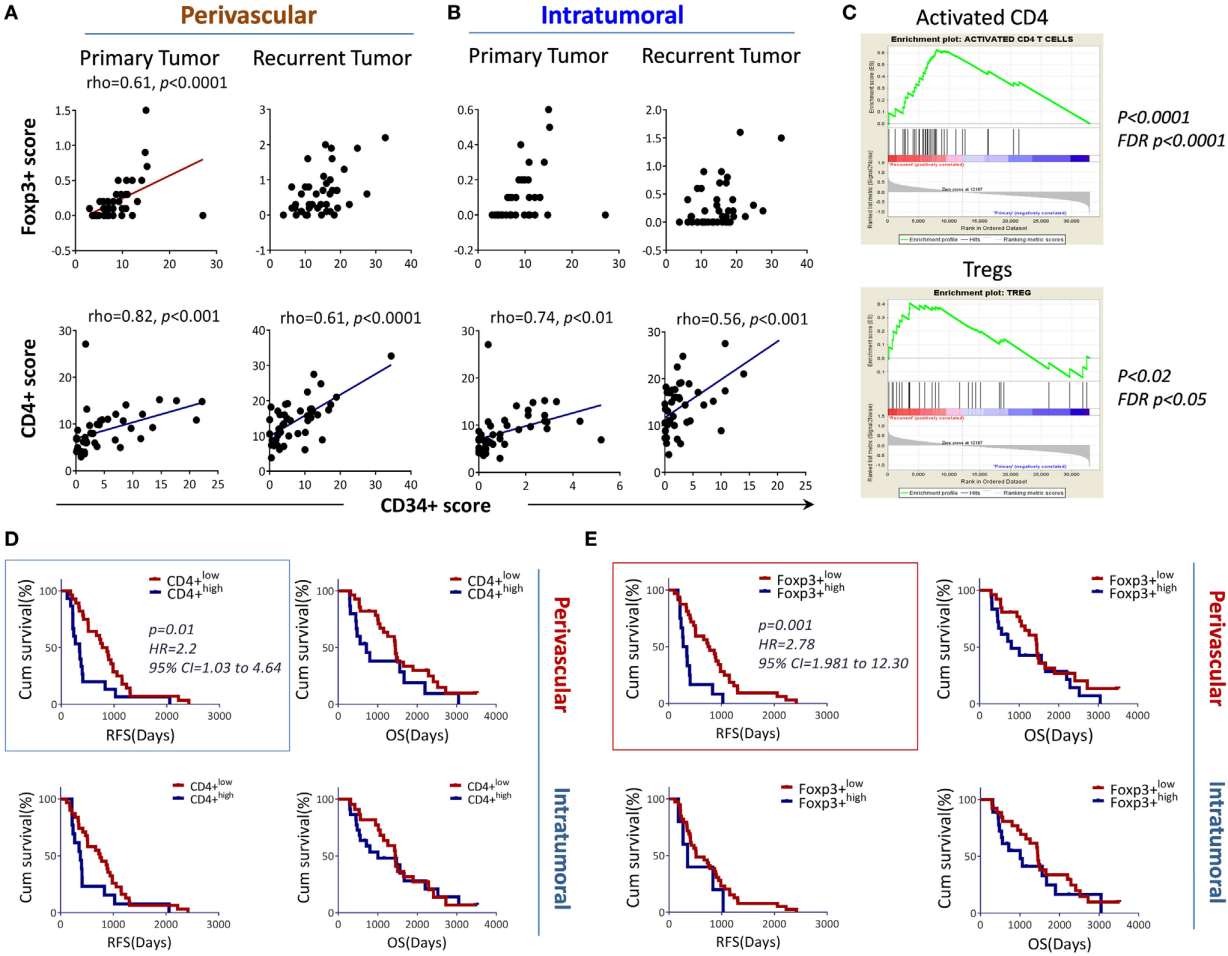
Perivascular Foxp3+ T cells associated with angiogenesis and tumor progression. **(A,B)** Perivascular Foxp3+ (upper panel) or CD4+ T cells (lower panel) were positively associated with CD34+ circles, respectively. **(C)** Regulatory T cells (Tregs) signature genes enriched by the tumor recurrence. The microarray data described in Figure S1 in Supplementary Material were input into Gene Set Enrichment Analysis (GSEA) for an analysis of activated CD4 T cells and Tregs. **(D)** Association of perivascular or intratumoral CD4+ T cells with RFS (left) and overall survival (OS) (right). **(E)** Association of intratumoral or perivascular Foxp3+ T cells with RFS and OS. The association between the markers and RFS, OS is shown using Kaplan–Meier survival curves, the median value was used for stratifying the low and high in each recurrent group. The log-rank test was used to compare the differences.

The association between tumor-infiltrating CD4+ T cells and tumor angiogenesis led us to further test the association between angiogenesis and a subset of immune inhibitory CD4+ T cells or regulatory T cells (Tregs). We found that perivascular Foxp3+ (Figures 5A–B upper panel), and CD4+ T cells (Figures 5A–B, lower panel) in the primary tumors correlated with tumor blood vessels. No similar trend was found for the CD8+ T cells in both geographic locations (data not shown). We sought to confirm the observation by using gene enrichment for Treg signature genes using paired samples. The results indicate that tumor recurrence significantly increases CD4 (similar to the results from bevacizumab resistant tumors) and Tregs signals in these tumors, whereas other cell subsets, i.e., NK, Th17, show contrasting results (Figure 5C and data not shown). To evaluate the correlation between differential geographic T cell subsets and clinical outcomes (RFS, OS), Kaplan–Meier survival curves were plotted using these tested parameters against RFS and OS. We found that only perivascular CD4+ T cells and Tregs were associated with shorter RFS (*p* = 0.007 for CD34, *p* = 0.01 for CD4+ T cells and *p* = 0.001 for Foxp3+ T cells). No association was observed for these factors with respect to OS (Figures 5D–E). Additionally, there was no association found for perivascular or intratumoral CD8+ T cells with respect to RFS and OS (Figure S3 in Supplementary Material). Next, to assess if key T cell subsets independently predict glioma progression, Cox multivariate regression analyses were performed (by including factors, such as age, sex, chemo/radiotherapy, CD34+ circles and perivascular/intratumoral infiltrating T cell subsets). The results concluded that only perivascular Foxp3+ T cells were found to be an independent predictor of shortened RFS (HR = 4.199, 95% CI 1.522–11.584, *p* = *0.006*) when all the patients were included.

## Discussion

Several clinical and experimental studies have evaluated the correlation between TILs and survival in patients with gliomas. Some reports indicate a positive correlation between the abundance of CD3+ or CD8+ TILs and patients survival (19, 20, 34, 35), whereas others report conflicting observations (36). For Tregs, no consensus has been reached for patients with gliomas (22, 35, 37). Nearly all the reports were based on analysis from un-paired patient populations. In this study, we collected 44 paired patient tumor samples and compared changes in gene expression before and after tumor recurrence. Compared to the other two groups, the most significant change in gene expression after recurrence was seen in the DA–GBM group (secondary GBM). Intriguingly, this group had the longest RFS, wider ranges of T cell infiltration in primary tumors, the greatest change in tumor blood vessel and TIL subsets post-recurrence, and the strongest correlation with the tumor vascularization, suggesting that disease progression in this group is unique. The kinetics of CD34+ vascular circles and the abundance of T cell subsets after recurrence showed an identical trend which led us to hypothesize that an interconnection exists between neovascularization and TILs. Indeed, we found a positive correlation between CD4+ TILs and the CD34+ circles, but not for CD8+ or CD3+ T cells (CD3+ T cell data not shown). To figure out why only CD4+ T cells correlated, we analyzed RNA-seq data of 155 primary GBMs culled from TCGA. In these tumors, CD4 expression was approximately 29-fold higher than CD8 transcripts (CD8A and B). This difference is not expected to be due to an error from sample preparation and analysis because the same analysis of other cancer types demonstrated higher expression of CD8 transcripts then CD4. The observation led us to speculate that the CD8+ T cells seeing in tumor tissues by IHC may be inadequate for the RNA-seq detection. Additionally, results from our recent study indicate that selective apoptosis of CD8+ T cells occurs in GBMs (38). Next, we utilized public data from Bevacizumab-treated paired patient samples; 15 patients had microarray data for their tumors before and after the bevacizumab treatment (31, 32). The results demonstrated that genes of activated CD4+ T cells were significantly increased in tumors after the recurrence, whereas other cell populations presented less or no change. These results confirmed our observation that CD4+ T cells are preferentially linked with tumor angiogenesis. Moreover, the gene profiles obtained by RNA-seq analysis of isolated CD4+ and CD8+ T cells also suggests that the activated CD4+ T cells express more pro-angiogenic molecules such as a CD40 ligand and Aquaporin 3 (39, 40). The evidence from previous reports supports our hypothesis. In an ischemia animal model, CD4-deficient mice have impaired capability to undergo angiogenesis (41). Although we were unable to be sure that FoxP3+ cells were CD4+ T cells by IHC staining (due to technical difficulties), we predict these cells to be Tregs based on: (1) morphology; (2) geographic location (i.e., perivascular association); (3) tumor immunosuppressive microenvironment limiting passage of activated T cells; and (4) gene enrichment studies demonstrating enhanced activated CD4+ cells and Tregs upon tumor recurrence. Ongoing studies in animal models have linked glioma angiogenesis and Treg involvement; combining VEGF and CD25 blockades restores the IFN-γ production by T cells previously suppressed by gliomas and significantly prolongs the OS in mice compared with single drug treatment (Long et al., manuscript in preparation). These data suggest that CD4+ T cells, including Tregs, may be involved in glioma angiogenesis.

T cell infiltration into the Virchow–Robin space is distinct from T cell infiltration into intratumoral spaces (42). Moreover, intratumoral TILs appear to associate with more favorable clinical outcomes (43). In gliomas, however, more investigations are necessary to draw definitive conclusions (30, 44). Our results showed that only one-third of all TILs are located in the intratumoral space while two-thirds of them (presumably CD4+ T cells, including Tregs) surrounding the tumor vessels. We found that Tregs highly correlated with the density of blood vessels in primary tumors, but only in the perivascular zone. Importantly, these perivascular Tregs cells were identified as an independent risk factor for tumor recurrence.

Recent genetic classification schemas have advanced our understanding of molecular characteristics and factors that impact tumor progression and survival (45–47). The status of IDH, *TERT* promoter mutations, and the deletion of chromosome 1p/19q was found to significantly impact patient clinical outcome (45, 48, 49). Beyond the scope of this report, we also examined our patients’ IDH mutation status; among the 44 patients, 22 tumors harbored the mutation, while the other 22 were IDH wild type (mostly from the GBM-GBM group). We uncovered that IDH mutations impact on the tumor immune landscape, and affect survival outcomes (Mu et al., manuscript under review). We included all patient data in Figure 5, and when patients in the GBM-GBM groups were removed, the main conclusion of Foxp3+ T cells as the independent risk factor for tumor recurrence remained true. Presumably, these Foxp3+ T cells are CD4 positive since it was the predominant T cell expressed transcript observed in GBM, and we also have found that CD8+ T cells are apoptotic in GBM (50). Thus, these CD4+ Foxp3+ T cells not only play the key role in pro-immunosuppression but also possess the pro-angiogenic function of the CD4+ T cells. The dual effects of these cells in primary tumors make them a strong player in the promotion of tumor progression in juxtaposition with the extremely low expression of CD8 transcripts in primary GBMs, which can be a major obstacle in tumor treatment.

In summary, only one-third of TILs were found in the intratumoral space with minimal expression of CD8 transcripts in primary tumors, thus limiting the overall strength of the antitumor response. The predominant population of CD4+ T cells may promote tumor angiogenesis, and in conjunction with perivascular CD4+ Tregs predispose tumor recurrence/progression in patients with gliomas.

## Author Contributions

Conception and design: LM, ZL, and JH. Development of methodology: LM, CY, QG, YL, HG, YC, LJ, JQ, JJi, JJiang, YG, JW, and YS. Data analysis: LM, CY, PK, and JH. Analysis and interpretation of data: LM, CY, ES, ZL, and JH. Writing, review, and revision of the manuscript: LM, YC, GL, ES, DM, ZL, and JH. Study supervision: JH.

## Conflict of Interest Statement

The authors declare that the research was conducted in the absence of any commercial or financial relationships that could be construed as a potential conflict of interest.

## Supplementary Material

Figure S1 Global gene profile changes and clinical outcomes for patients with DA to DA (DA-DA); DA to GBM (DA-GBM) and GBM to GBM (GBM-GBM) recurrence.

Figure S2 Representative images of CD34+ circles and intratumoral and perivascular CD3+, CD4+, CD8+, and Foxp3+ TIL cell subsets in 3 recurrent groups.

Figure S3 No association between CD8+ T cells with RFP in primary tumors.

Table S1 Upregulated genes after DA-GBM.

Table S2 Patients’ demographic and clinical characteristics.

Table S3 Microarray analysis of paired surgical resected tumors (3 pairs DA to DA, 3 pairs GBM to GBM and 3 pairs DA to GBM).
