# Supplementary figures and images for: CD4+ and Perivascular Foxp3+ T Cells in Glioma Correlate with Angiogenesis and Tumor Progression
Source: Front Immunol. 2017 Nov 7;8:1451. doi: 10.3389/fimmu.2017.01451 (PMC5673996; doi:10.3389/fimmu.2017.01451)

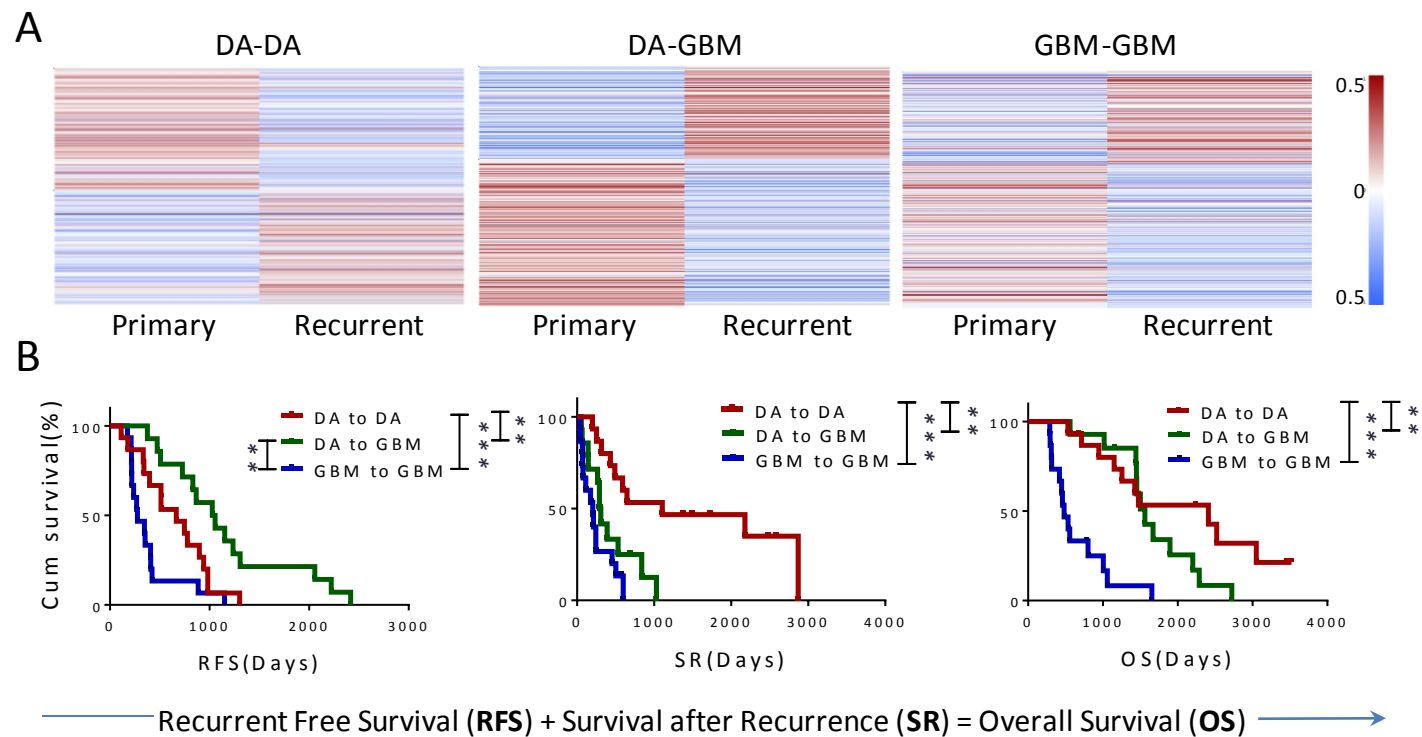

Supplement: Figure S1 — Global gene profile changes and clinical outcomes for patients with DA to DA (DA-DA); DA to GBM (DA-GBM) and GBM to GBM (GBM-GBM) recurrence. [file Data_Sheet_1.PDF]

# DA-DA group

A

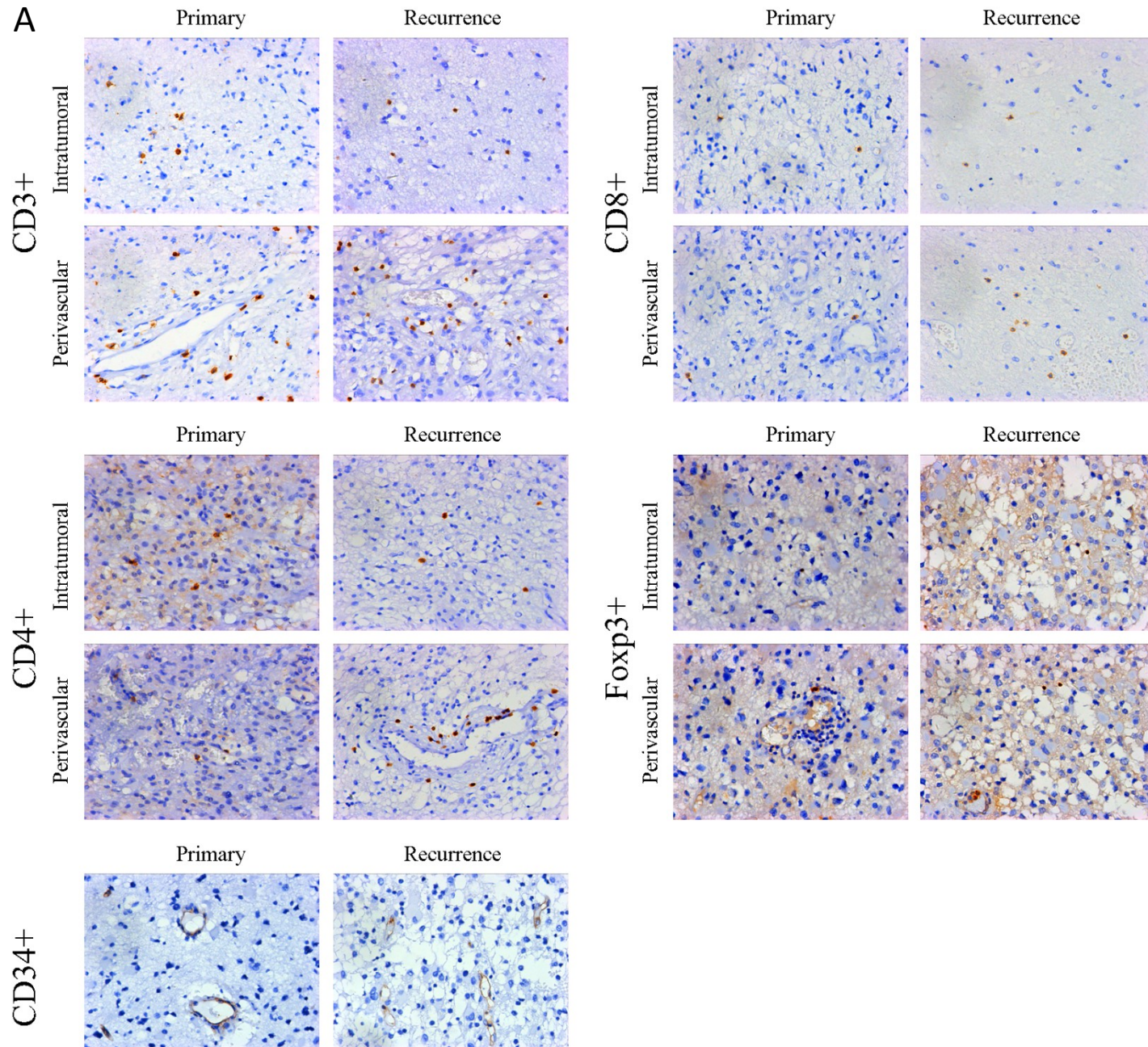

## DA-GBM group

B

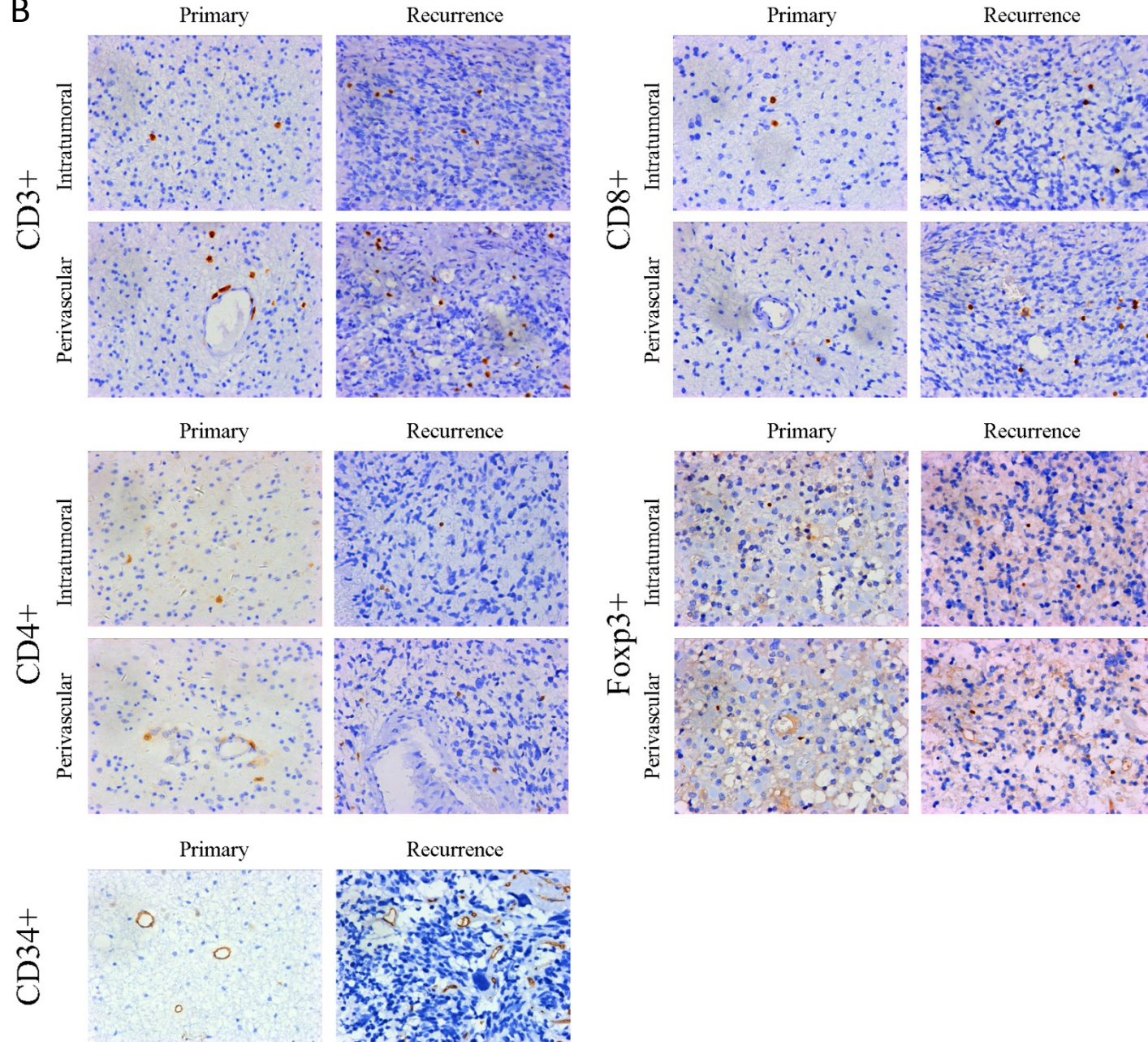

# GBM-GBM group

C

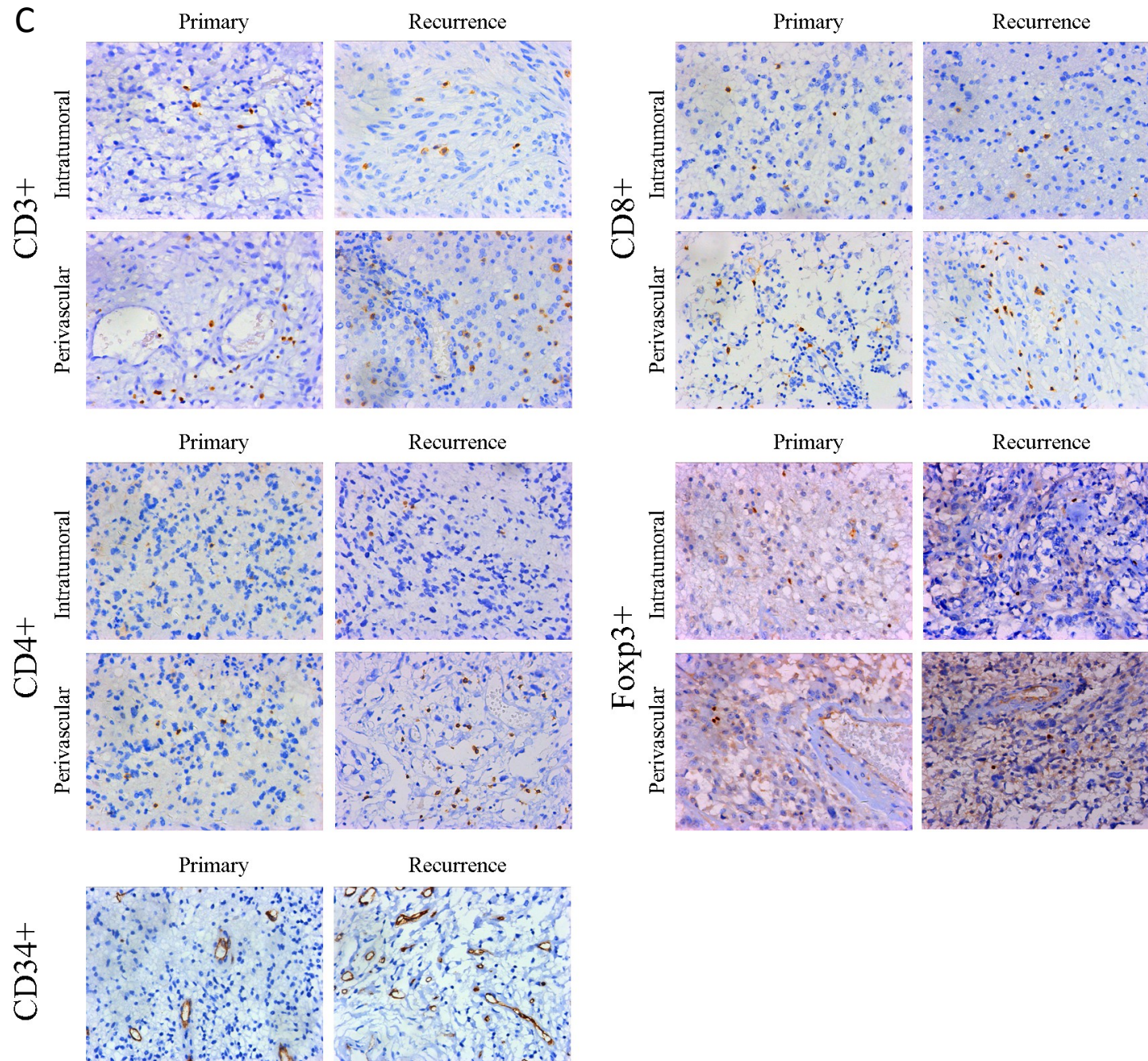

Supplement: Figure S2 — Representative images of CD34+ circles and intratumoral and perivascular CD3+, CD4+, CD8+, and Foxp3+ TIL cell subsets in 3 recurrent groups. [file Data_Sheet_2.PDF]

A

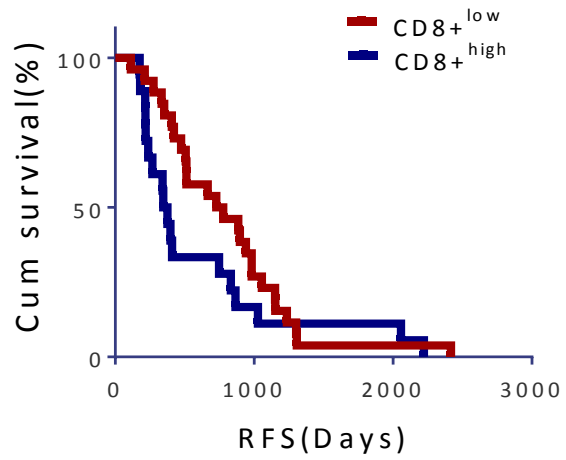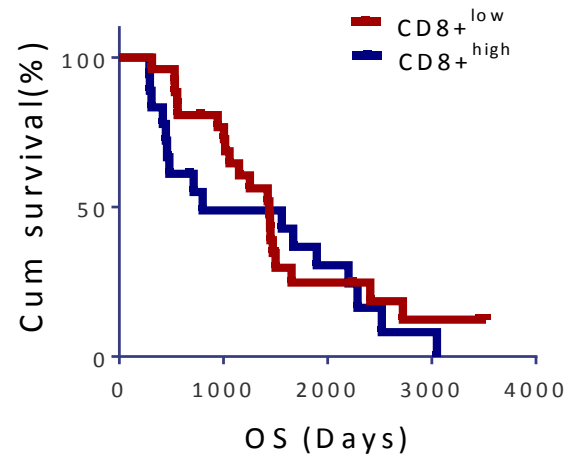

Perivascular

B

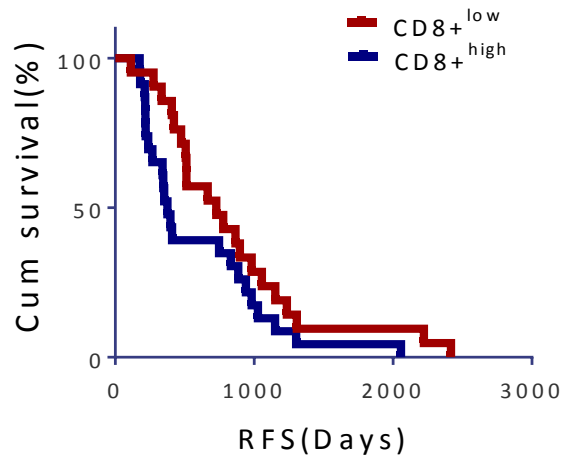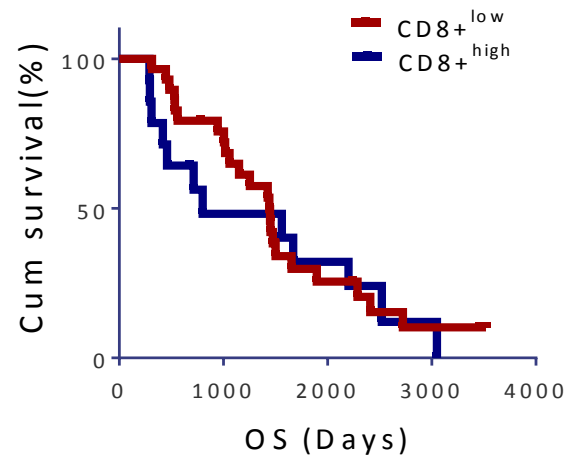

Intratumoral

Supplement: Figure S3 — No association between CD8+ T cells with RFP in primary tumors. [file Data_Sheet_3.PDF]
