# Supplementary material for: CD4+ and Perivascular Foxp3+ T Cells in Glioma Correlate with Angiogenesis and Tumor Progression
Source: Front Immunol. 2017 Nov 7;8:1451. doi: 10.3389/fimmu.2017.01451 (PMC5673996; doi:10.3389/fimmu.2017.01451)
Supplement: Table S1 — Upregulated genes after DA-GBM. [file Table_1.PDF]

Supplementary Table 1 Upregulated genes after DA-GBM

Genes	P-Value	Fold Change
VEGFA	0.021	10.3
UBE2C	0.013	10.2
HELLS	0.026	8.1
PDLIM1	0.045	7.8
ATF3	0.002	7.7
MYBL2	0.042	7.7
CRNDE	0.049	6.9
MAD2L1	0.046	6.7
CCNB2	0.007	6.5
PTTG1	0.048	5.8
SLC2A3	0.032	5.7
CCNB1	0.037	5.2
TROAP	0.024	5.0
CCNA2	0.020	4.5
RAD51AP1	0.028	4.4
LMO7	0.031	4.4
CDK2	0.042	4.0
MKI67	0.047	4.0
SOX11	0.042	3.9
PDE7A	0.013	3.8
