# Supplementary material for: CD4+ and Perivascular Foxp3+ T Cells in Glioma Correlate with Angiogenesis and Tumor Progression
Source: Front Immunol. 2017 Nov 7;8:1451. doi: 10.3389/fimmu.2017.01451 (PMC5673996; doi:10.3389/fimmu.2017.01451)
Supplement: Table S2 — Patients’ demographic and clinical characteristics. [file Table_2.PDF]

Supplementary Table 2. Patients demographic and clinical characteristics

Characteristic	DA to DA (WHO grade II-II) (n=15)		GBM to GBM (WHO grade IV-IV) (n=15)		DA to GBM (WHO grade II-IV) (n=14)	
	No.	%	No.	%	No.	%
**Age at primary surgery, years**						
Mean±SE	42.9 ± 8.7		45.9±13.4		40±8.3	
Range	30-58		22-68		29-53	
**Age at recurrent surgery, years**						
Mean±SE	44.6±8.7		46.7±13.8		43±7.8	
Range	31-59		23-69		31-55	
**Sex**						
Male	4	26.7	6	40	5	35.7
Female	11	73.3	9	60	9	64.3
**Race/ethnicity**						
Han	14	92.9	15	100	14	100
Mongolian	1	7.1				
**Laterality**						
Left	9	60	11	73.3	11	78.6
Right	6	40	4	26.7	3	21.4
**Tumor Location**						
Frontal	6	40	6	40	7	50
Temporal	5	33.4	2	13.3	5	35.7
Parietal	2	13.3	4	26.7	2	14.3
Occipital	2	13.3	3	20	0	0
**Tumor size in primary surgery, cm^3**						
Mean±SE	35.96±12.09		40.88±11.5		32.14±10.97	
Range	16.66-56.88		18.56-64.14		12-48	
**Extent of resection in primary surgery**						
Gross total resection	15	100	14	93.3	13	92.8
Subtotal resection (50-99%)			1	6.7	1	7.2
**Adjuvant therapy after primary surgery**						
Radiotherapy only	10	66.7	2	13.3	8	57.1
Chemotherapy only	0		0		0	
Radiotherapy plus Chemotherapy	5	33.3	13	86.7	6	42.9
**First presenting symptom**						
Headache	5	33.3	8	53.3	6	42.9
Mental status change	1	6.7	0		1	7.1
Motor or movement change	4	26.7	4	26.7	1	7.1
Seizure/Epilepsy	3	20	0		3	21.4
Sensory or visual change	2	13.3	3	20	3	21.4
**Tumour size in recurrent surgery, cm^3**						
Mean±SE	37.36±17.75		50.43±19.93		43.68±17.53	
Range	15-80.3		30-110.01		20.5-85	
**Mortality rate**						
Death	10	66.7	14	93.3	12	85.7
**Recurrence-Free Survival (RFS), days**						
Mean±SE	645.9±340.8		386.3±273.3		1161.2±648.3	
Range	113-1303		175-1150		375-2417	
**Overall survival (OS), days**						
Mean±SE	1547.7±836.2		627±378.2		1646.6±577	
Range	533-3050		309-1654		562-2724	
